# Cytokine and neuropeptide levels are associated with pain relief in patients with chronically painful total knee arthroplasty: a pilot study

**DOI:** 10.1186/s12891-016-1375-2

**Published:** 2017-01-14

**Authors:** Jasvinder A. Singh, Siamak Noorbaloochi, Keith L. Knutson

**Affiliations:** 1 Medicine Service, VA Medical Center, 700 19th St S, Birmingham, AL 35233 USA; 2 Department of Medicine at School of Medicine, University of Alabama at Birmingham Faculty Office Tower, 805B, 510 20th Street S, Birmingham, AL 35294 USA; 3 Division of Epidemiology at School of Public Health, University of Alabama at Birmingham, 1720 Second Ave. South, Birmingham, AL 35294-0022 USA; 4 Department of Orthopedic Surgery, Mayo Clinic College of Medicine, 200 1st St SW, Rochester, MN 55905 USA; 5 Department of Immunology, Mayo Clinic College of Medicine, 200 1st St SW, Rochester, MN 55905 USA; 6 Center for chronic disease Outcomes Research, Minneapolis Veterans Affairs Health are System Center, Minneapolis, MN 55121 USA; 7 Department of Medicine, University of Minnesota, 401 East River Parkway, Minneapolis, MN 55455 USA

**Keywords:** Cytokine, Substance P, Pain, Primary total knee arthroplasty, TKA

## Abstract

**Background:**

There are few studies with an assessment of the levels of cytokines or neuropeptides as correlates of pain and pain relief in patients with painful joint diseases. Our objective was to assess whether improvements from baseline to 2-months in serum cytokine, chemokine and substance P levels were associated with clinically meaningful pain relief at 2-months post-injection in patients with painful total knee arthroplasty (TKA).

**Methods:**

Using data from randomized trial of 60 TKAs, we assessed the association of change in cytokine/chemokine/Substance P levels with primary study outcome, clinically important improvement in Western Ontario McMaster Osteoarthritis Index (WOMAC) pain subscale at 2-months post-injection using Student’s t-tests and Spearman’s correlation coefficient (non-parametric). Patients were categorized as pain responders (20-point reduction or more on 0-100 WOMAC pain) vs. pain non-responders. Sensitivity analysis used 0–10 daytime pain numeric rating scale (NRS) instead of WOMAC pain subscale.

**Results:**

In a pilot study, compared to non-responders (*n* = 23) on WOMAC pain scale at 2-months, pain responders (*n* = 12) had significantly greater increase in serum levels of IL-7, IL-10, IL-12, eotaxin, interferon gamma and TNF-α from baseline to 2-months post-injection (*p* < 0.05 for all). Change in several cytokine/chemokine and substance P levels from pre-injection to 2-month follow-up correlated significantly with change in WOMAC pain with correlation coefficients ranging −0.37 to −0.51: IL-2, IL-7, IL-8, IL-9, IL-16, IL-12p, GCSF, IFN gamma, IP-10, MCP, MIP1b, TNF-α and VEGF (*n* = 35). Sensitivity analysis showed that substance P decreased significantly more from baseline to 2-months in the pain responders (0.54 ± 0.53; *n* = 10) than in the pain non-responders (0.48 ± 1.18; *n* = 9; *p* = 0.023) and that this change in serum substance P correlated significantly with change in daytime NRS pain, correlation coefficient was 0.53 (*p* = 0.021; *n* = 19). Findings should be interpreted with caution, since cytokine analyses were performed for a sub-group of the entire trial population.

**Conclusion:**

Serum cytokine, chemokine and Substance P levels correlated with pain response in patients with painful TKA after an intra-articular injection in a randomized trial.

## Background

Total knee arthroplasty (TKA) is one of the most successful procedures performed in patients with end-stage osteoarthritis (OA) and other arthritides with refractory pain [1]. While most patients report improved pain and function outcomes after TKA, persistent pain is reported by 6.5% or more patients post-TKA [2].

The role that cytokines and neurotransmitters play in joint pain is of great interest and has been the focus of recent research [3]. In an animal model of OA, cytokine levels were higher in OA joints, which were reduced by treatment with coenzyme Q-10 that also led to significant pain relief [4]. TNF-α levels were associated with WOMAC pain scores in patients with OA [5]. Joint fluid substance P (SP) levels in knee joints were elevated in painful knee joints with OA [6] and in preoperative joint fluid in those with greater pain relief after knee arthroplasty [6]. Studies showed that  higher levels of cytokines such as interleukin-8 (IL-8), IL-6 and tumor necrosis factor-alpha (TNF-α) were associated with implant loosening in patients with joint arthroplasty  [7, 8]. To our knowledge, there are limited or no data regarding the role of cytokines in pain severity in patients with painful TKA or relief with interventions.

We examined the data from a randomized trial of patients with persistently painful TKA [9] to test the hypotheses whether in patients with painful TKA, change (baseline to 2-months) in serum cytokine, chemokine and substance P (neuropeptide) levels was associated with clinically meaningful pain relief at 2-months post-injection. We also assessed whether baseline to 2-month change in cytokine/chemokine/substance P level correlated with change in pain severity from baseline to 2 months.

## Methods

### Clinical trial population

We used the data from patients enrolled in a previously published 6-month, 2-arm, parallel group, blinded placebo-controlled randomized trial (NCT00403273), details provided elsewhere [9]. Briefly, 60 TKAs were randomized in 1:1 to single intra-articular injection of active (onabotulinum toxin A) vs. placebo (saline) to assess its short-term anti-nociceptive efficacy. The research pharmacist prepared computerized randomization with permuted blocks of four patients each and prepared the treatment and placebo syringes using a strict standardized protocol. Both placebo and onabotulinum toxin A injections were transparent and could not be differentiated. In this triple-blind study, patients, investigators (PI, blinded investigators performing assessments, research associates) and the statistician were blinded (all analyses were completed and tables finalized before the pharmacist revealed the designation for group 1 vs. group 2). The PI injected the affected TKA joint using the standardized medial or lateral approach [10, 11]. This study was adequately powered with 19 patients/group were needed for 80% power and 24 patients/group for 90% power (assuming 25% loss to follow-up), to detect a difference of 43% in proportion of patients reporting a clinically meaningful improvement in pain, based on previously published studies [12, 13].

Primary outcome assessment was at 2-months. A protocol modification to collect blood specimens to understand the mechanism of pain relief in patients with persistently painful TKA was made around the mid-point of patient recruitment upon receipt of federal funds, which provided resources to perform these assays, which were not available previously [9]. The Institutional Review Board (IRB) at the Minneapolis VA approved the study.

### Serum cytokine and neuropeptide assays

Specimens were drawn both at baseline pre-injection and 2-month post-injection. We performed the serum cytokine assay using the BioRad human 27-plex cytokine panel (Cat # 171-A11127, Bio-Rad, San Diego CA; Table 1; http://www.bio-rad.com/en-jp/sku/m500kcaf0y-bio-plex-pro-human-cytokine-27-plex-assay). 100 μl of Bio-Plex assay buffer was added to each well of a Multiscreen MABVN 1.2 μm microfiltration plate followed by the addition of 50 μl of the multiplex bead preparation. Following washing of the beads with the addition of 100 μl of wash buffer, 50 μl of the samples or the standards was added to each well and incubated with shaking for 30 min at room temperature. Standard curves were generated with a mixture of 27 cytokine standards and eight serial dilutions ranging from 0 to 32,000 picogram/ml. The plate was then washed three times followed by incubation of each well in 25 μl of pre-mixed detection antibodies for 30 min with shaking. The plate was further washed and 50 μl of streptavidin solution were added to each well and incubated for 10 min at room temperature with shaking. The beads were given a final washing and re-suspended in 125 μl of Bio-Plex assay buffer. Cytokine levels in the sera were quantitated by analyzing 100 μl of each well on a Bio-Plex using Bio-Plex Manager software version 4.0.

**Table 1:** Components of the 27-cytokine panel

Interleukins (IL): IL-1 beta, IL-1 alpha, IL-2, IL-4, IL-5, IL-6, IL-7, IL-8, IL-9, IL-10, IL-12 (p70), IL-13, IL-15, IL-17
Basic fibroblast growth factor (FGF)
Eotaxin
granulocyte colony stimulating factor (G-CSF), granulocyte macrophage colony stimulating factor (GM-CSF)
Interferon gamma (IFN- γ), Interferon gamma-induced protein 10 (IP-10)
Monocyte chemoattractant protein-1 (MCP-1)
Macrophage Inflammatory Protein-1 alpha (MIP-1α), MIP-1 beta (MIP-1β)
Platelet-derived growth factor (PDGF)
Regulated upon Activation, Normal T cell Expressed and presumably Secreted (RANTES)
Tumor necrosis factor-alpha (TNF-α)
Vascular endothelial growth factor (VEGF)

Serum neuropeptide assay included enzyme linked immunoassays for Substance P (SP), (R&D Systems, Minneapolis, MN; https://www.rndsystems.com/products/substance-p-parameter-assay-kit_kge007#product-details), which was performed as per manufacturer’s instructions in a standard fashion. Samples, standards and controls were placed in a 96 well plate pre-coated with a polyclonal antibody. Primary antibody (for SP) solution and SP conjugated to horseradish peroxidase, were added to the wells and the plate was incubated overnight at 4° Celsius. After washing, substrate solution was added to the wells, incubated for 30 min, at room temp, in the dark and stop solution was added. The optical density of wells was determined using a micro plate reader set to 450 nm, with a reference wavelength of 570 nm.

### Statistical analyses

Change in serum cytokine, chemokine and Substance P levels were calculated as baseline minus 2-month levels for each patient, where specimen was available. We decided a priori not to impute any values. We defined clinically important improvement in pain as the following: WOMAC pain scale as 20-point absolute improvement or more (range 0–100; higher = more pain; main analysis); or 2-point reduction or more in index TKA daytime numeric rating scale (NRS) pain severity on 0–10 scale (higher = more pain) at 2-month post-injection (sensitivity analysis), as previously published [14]. Patients were categorized as pain responder (achieved clinically important improvement in WOMAC pain) vs. pain non-responders (did not achieve this clinically improvement in WOMAC pain). We used student’s t-tests to compare the mean change in cytokine chemokine and Substance P levels baseline to 2-months post-injection between pain responders and non-responders. We assessed non-parametric Spearman’s correlation between cytokine/neurotransmitter level change from baseline to 2-months and the decrease in WOMAC pain (main analysis) or 0–10 daytime pain numeric rating scale (NRS; sensitivity analyses), baseline to 2-months, to account for non-normal distributions.

## Results

Patients in the randomized trial had a mean age of 67 years, 84% were male and 96% were Caucasian. The mean duration of TKA pain was 4.5 years (SD, 4.8) and 75% were primary TKAs.

Compared to non-responders (*n* = 23) on WOMAC pain scale at 2-months, pain responders (*n* = 12) had significantly greater increase in serum levels of IL-7, IL-10, IL-12 (p70), eotaxin, IFN- γ and TNF-α from baseline to 2-months post-injection (*p* < 0.05 for all; Table 2). Several other cytokines showed a non-significant trend by pain-responder status (*p* ≤0.32; Table 2). In sensitivity analysis in a smaller set of patients, who reported daytime NRS pain on 0–10 scale, serum substance P decreased significantly more in the pain responders (0.54 ± 0.53; *n* = 10) than in the pain non-responders (0.48 ± 1.18; *n* = 9; *p* = 0.023) 2-months post-injection.

**Table 2:** Association of change in serum cytokine and neurotransmitter levels from baseline to 2-months with pain responder status on WOMAC pain at 2-month post-injection in painful TKA

	WOMAC Pain Responder*	N	Mean Change (FU-Baseline)	Std. Deviation	Std. Error Mean	*P*-value
Interleukin (IL)-7	No	23	0.084	0.91	0.19	**0.01**
	Yes	12	1.07	1.17	0.34	
IL-10	No	23	8.51	20.90	4.36	**0.01**
	Yes	12	27.72	21.56	6.22	
IL-12 p70	No	23	3.36	8.17	1.70	**0.004**
	Yes	12	12.91	9.60	2.77	
Eotaxin	No	23	−2.03	13.92	2.90	**0.046**
	Yes	12	7.85	12.28	3.54	
Interferon gamma (IFN-γ)	No	23	−1.24	23.18	4.83	**0.03**
	Yes	12	15.61	13.35	3.85	
Tumor necrosis factor-alpha (TNF-α)	No	23	4.46	21.32	4.44	**0.03**
	Yes	12	22.22	24.65	7.12	
IL-4	No	23	−0.02	0.16	0.03	0.12
	Yes	12	0.06	0.12	0.03	
IL-6	No	23	−15.90	275.66	57.48	0.09
	Yes	12	137.35	182.09	52.56	
IL-13	No	23	4.90	13.25	2.76	0.15
	Yes	12	12.18	14.87	4.29	
IL-15	No	23	0.31	11.87	2.47	0.09
	Yes	12	7.36	10.52	3.038	
Macrophage Inflammatory Protein-1 beta (mip1b)	No	23	5.55	91.43	19.06	0.16
	Yes	12	53.35	96.39	27.82	
Substance P	No	14	0.08	1.11	0.298	0.32
	Yes	5	−0.46	0.61	0.273	

Only those associations that either had a significant *p*-value or a *p*-value <0.33 are listed; The levels of other cytokine did not differ significantly between pain responders and pain non-responders (IL-1 beta, IL-1 alpha, IL-2, IL-5, IL-8, IL-9, IL-17, Basic FGF, G-CSF, GM-CSF, IP-10, MCP-1, MIP-1 alpha, PDGF, RANTES, and VEGF). Significant *p*-values <0.05 are in bold

Positive changes mean that at the 2-month follow-up time, the levels were higher than the baseline and a negative sign means follow-up levels were lower

WOMAC Pain Responder* is defined as reduction in WOMAC pain subscale of 20 or more 0–100 scale

Change in several cytokine and chemokine levels from pre-injection to 2-month follow-up correlated significantly with change in WOMAC pain with correlation coefficients ranging −0.37 to −0.51: IL-2, IL-7, IL-8, IL-9, IL-16, IL-12 (p70), GCSF, IFN- γ, IP-10, MCP, MIP1b, TNF-α and VEGF (*n* = 35; Table 3). In sensitivity analysis, we additionally noted a change in serum substance P from pre-injection to 2-month follow-up correlated significantly with change in daytime NRS pain, correlation coefficient was 0.53 (*p* = 0.021; *n* = 19).

**Table 3:** Non-parametric correlation of change in serum cytokine and neurotransmitter levels from baseline to 2-months with change in WOMAC pain at 2-month post-injection in painful TKA, showing statistically significant associations

Baseline to 2-month change in serum levels*	Spearman’s correlation coefficient	*P*-value
IL2	−0.37	0.03
IL7	−0.42	0.01
IL8	−0.51	**0.002**
IL9	−0.50	**0.002**
IL16	−0.48	**0.004**
IL12p70	−0.56	**<0.001**
GCSF	−0.34	0.04
IFN gamma	−0.48	**0.003**
IP10	−0.38	0.02
MCP	−0.35	0.03
MIP1b	−0.49	**0.003**
TNF-alpha	−0.42	0.01
VEGF	−0.38	0.02

*Data from 35 patients were available

Only those cytokines that had a significant *p*-value <0.05 are listed in bold; other cytokines were not significantly associated with change in WOMAC pain at 2-month post-TKA

## Discussion

In this ancillary pilot study, we assessed whether changes in serum levels of cytokines, chemokines and Substance P from baseline to 2-month post-injection were associated with clinically meaningful pain relief at 2-month post-injection. We performed a mechanistic study and used data from our randomized study of intra-articular injection for painful TKA [9]. In many patients, the pain improvement lasted through the 6-month follow-up period, indicating that the joint pain relief was somewhat durable [9]. We found that several cytokine levels, including IL-7, IL-10, IL-12 (p70), eotaxin, IFN- γ and TNF-α, changed significantly more in WOMAC pain responders compared to pain non-responders (responders defined as those with pain decrement of 20 points or more on 0-100 scale). Correlation analyses identified additional cytokines with moderate correlations with WOMAC pain scores (baseline to 2-month change in cytokine level with baseline to 2-month change in WOMAC pain) in addition to these, including IL-2, IL-8, IL-9, IL-16, GCSF, IP-10, MCP, MIP1b and VEGF. In sensitivity analysis using a smaller dataset, serum substance P level reduction was greater in pain responders using the 0–10 daytime NRS pain than pain non-responders (pain decrement of two points or more o 0–10 scale). The direction and magnitude were similar to the responder analysis by WOMAC pain, however, standard deviations were larger in WOMAC pain analysis, leading to the difference in substance P levels being non-significant (*p* = 0.32) in the main analysis, but significant in sensitivity analysis (*p* = 0.023). This is a hypothesis-generating study and therefore, these findings need to be replicated in future studies.

The traditional view of cytokines/chemokines being either anti- or pro-inflammatory [15] has been challenged [16], since they can serve either role depending on the condition and the body organ. Higher levels of pro-inflammatory cytokines have been linked to pain [17–20]. On the other hand, change in pro- and anti-inflammatory cytokines with treatment in other pain conditions do not map precisely to their associations with pain condition at baseline [21–24]. Studies that have investigated the potential mechanisms of joint pain relief in intervention studies are lacking. Such studies can provide insights into mechanisms of action of an intervention, and discover mediators of joint pain relief in patients with OA. Our study begins to fill this knowledge gap by providing data among patients with painful joint arthroplasty.

Other recent uncontrolled studies have documented a potential role of cytokines, chemokines and Substance P in the failure of total joint replacement. In a recent study, both IL-1 beta and IL-2 levels were significantly lower (*p* < 0.025) in 10 patients with stable, painless, well-functioning, cemented total knee or hip arthroplasties (TKA/THA) than patients with aseptically loosened, painful, arthroplasties [25]. Genetic variants of pro-inflammatory cytokines TNF-alpha and IL-6 were associated with susceptibility to severe osteolysis after THA, a condition that is associated with increasing pain and functional limitation [7]. Compared to OA, aseptic loosening of TKA was associated with up-regulated expression of several cytokines including IL-8 and MMP9 and low levels of inflammatory cytokines [8].

In patients undergoing revision surgery for painful primary hip arthroplasty, nerve fibers with positive immunostaining to Substance P were found in bone-prosthesis interface membranes [26]. Joint fluid Substance P levels were elevated in painful knee joints with osteoarthritis that underwent TKA, but not in normal/asymptomatic contralateral knees [6]. Significantly greater pain relief after knee arthroplasty was seen in patients with an elevated preoperative joint fluid Substance P level compared to patients with normal Substance P levels [6]. In an animal model of OA, MMP-13, IL-1b, IL-6 and IL-15 were up-regulated in OA joints and the levels were reduced by treatment with coenzyme Q-10 that led to significant pain relief [4]. In a study of 47 OA patients, TNF-alpha levels were associated with WOMAC pain and overall scores [5]. Thus, cytokine levels have been correlated with OA and arthroplasty joint pain in observational studies. Our study provides evidence showing that a change in cytokine levels correlated with pain relief in patients with painful TKA in a clinical trial who had a clinically meaningful pain relief after an intra-articular injection.

Our study findings must be interpreted considering study limitations. The sample size for this ancillary study was small. A protocol modification was made to the main study on receipt of federal funding that allowed us to collect specimens and perform analyses, and therefore these analyses were performed on a subset of the main trial, since almost 40% of the study cohort had already been enrolled in the study. Therefore, we likely missed several important findings due to type II error. Subsequent studies should enroll a larger number of patients to explore this important aspect of treatment for patients with painful arthroplasty. We examined the patients in the pain responder vs. non-responder categories, as specified a priori in our analytic plan. Since our hypothesis was to assess the cytokine, chemokine and neuropeptide mediators of joint pain relief in painful arthroplasty, we limited our analyses by whether patient was or was not a responder by clinically meaningful pain relief at 2-months post-injection. We adopted this approach rather than comparing Botulinum toxin group vs. placebo group, since we only had samples on half of the study participants in the RCT and both groups of patients with samples consisted of a mix of pain responders and non-responders, leading to an inadequate sample size for this comparison. We believe that the associations we noted are unlikely to be drug-specific, since responders included patients from both intervention and control arms. This analysis addressed our main objective to better understand the mechanism of pain improvement in patients who have had a failed TKA, and are undergoing a medical treatment to improve pain. Study strengths include the randomized, blinded study design, use of paired samples and focus on a condition that has significant public health impact. The presence of significant associations, even under the stringent categorization (responders/non-responders), with the current sample size, supports our underlying theoretical framework that cytokine, chemokine and substance P level changes after an intra-articular injection might be associated with pain relief.

## Conclusions

In conclusion, our exploratory study shows that patients who experience pain relief after an intra-articular injection have a different pattern of change in serum cytokine/chemokine levels than patients without pain relief. Our study identifies several cytokines that might play a role in relief of joint pain with local therapies. Our study has generated interesting hypotheses of mechanisms of pain relief in patients with painful TKA. Future studies need to confirm our findings in patients with painful arthroplasty, with similar intra-articular interventions, but also other dissimilar interventions, since pain pathways/mechanisms may be similar.

## Abbreviations

NRS: Numeric rating scale
OA: Osteoarthritis
TKA: Total knee arthroplasty
WOMAC: Western Ontario and McMaster Universities Osteoarthritis Index
